# Improving the Reliability of the Pavlovian Go/No-Go Task for Computational Psychiatry Research

**DOI:** 10.5334/cpsy.127

**Published:** 2025-12-18

**Authors:** Samuel Zorowitz, Gili Karni, Natalie Paredes, Nathaniel Daw, Yael Niv

**Affiliations:** 1 Princeton Neuroscience Institute, Princeton University, USA; 2 Department of Psychology, University of California, San Diego, USA; 3 Department of Psychology, Princeton University, USA

**Keywords:** Pavlovian go/no-go task, Pavlovian bias, reinforcement learning, reliability

## Abstract

**Background:**

The Pavlovian go/no-go task is commonly used to measure individual differences in Pavlovian biases and their interaction with instrumental learning. The task has also been widely used in computational psychiatry research, to correlate Pavlovian biases with mental health symptoms. However, prior research has reported unacceptable reliability for computational model-based performance measures for this task, limiting its usefulness in individual-differences research. Here, we apply several strategies previously shown to enhance task-measure reliability (e.g., task gamification, hierarchical Bayesian modeling for model estimation) to the Pavlovian go/no-go task, to improve the reliability of the task as a tool for future research.

**Methods:**

In two experiments, two independent samples of adult participants (N = 103, N = 110) completed a novel, gamified version of the Pavlovian go/no-go task multiple times over several weeks. We used hierarchical Bayesian modeling to derive reinforcement learning model-based indices of participants’ task performance, and to estimate the reliability of these measures.

**Results:**

In Experiment 1, we observed considerable practice effects, with most participants reaching near-ceiling levels of performance with repeat testing. Consequently, the test-retest reliability of some model parameters was unacceptable (as low as 0.379). In Experiment 2, participants completed a modified version of the task designed to lessen these practice effects. We observed greatly reduced practice effects and improved estimates of the test-retest reliability (range: 0.696–0.989).

**Conclusion:**

The results demonstrate that model-based measures of performance on our modified Pavlovian go/no-go task can reach levels of reliability sufficient for use in individual-differences research. We therefore provide the task code for use by the computational psychiatry community (as well as other researchers). Additional investigation is necessary to validate the modified version of the task in other populations and settings.

## Introduction

Humans (and other animals) have an innate tendency to approach rewarding stimuli and shrink from punishing stimuli (Carver & White, 1994). Depending on the context, these hardwired Pavlovian biases can either benefit or interfere with instrumental (i.e., action-outcome) learning. This is epitomized in the Pavlovian go/no-go task in which the required action (Go, No-Go) and outcome valence (reward, punishment) are orthogonalized (Guitart-Masip et al., 2012, 2014). In the task, participants are typically faster to learn actions that are congruent with Pavlovian response biases (i.e., a “Go” response to receive reward and a “No-Go” response to avoid punishment) as compared to Pavlovian-instrumental incongruent responses (i.e., inhibit action to receive reward, initiate action to avoid punishment).

The Pavlovian go/no-go task has been used in a large number of studies to probe individual differences in reward and punishment learning, of which many have reported changes in Pavlovian biases as a function of psychiatric conditions. For example, an increased tendency towards passive avoidance has been observed in individuals with generalized and social anxiety (Mkrtchian et al., 2017; Peterburs, Albrecht & Bellebaum, 2021), whereas active avoidance is amplified in individuals with a history of suicidal thoughts or behaviors (Millner et al., 2019). Pavlovian biases are larger in individuals with trauma exposure (Ousdal et al., 2018) and first-episode psychosis (Montagnese et al., 2020), but attenuated in individuals with depression (Huys et al., 2016) and schizophrenia (Albrecht et al., 2016). Pavlovian biases have also been associated with individual differences in personality (e.g., impulsivity; Eisinger et al., 2020) and genetics (Richter et al., 2014, 2021). In developmental and lifespan research, Pavlovian biases have been shown to exhibit a U-shape, decreasing from childhood to young adulthood and increasing again in older age (Betts et al., 2020; Raab & Hartley, 2020). At a finer temporal scale, Pavlovian biases are also reportedly modulated by state effects including mood (Weber et al., 2022), anger (Wonderlich, 2020), stress (de Berker et al., 2016), and fear (Mkrtchian, Roiser & Robinson, 2017).

However, three independent studies found that descriptive and computational-model based measures of performance on the Pavlovian go/no-go task exhibited low test-retest reliability over short (two-week) and long (6-, 18-month) retest intervals (Moutoussis et al., 2018; Pike et al., 2022; Saeedpour et al., 2023). Specifically, Moutoussis et al. (2018) reported Spearman correlations ranging from 0.10 to 0.43 over 6–18 month intervals, with the Pavlovian bias parameter showing particularly weak stability (*ρ* = 0.10, *p* = 0.017); Pike et al. (2022) reported correlations ranging from 0.18 to 0.495 for task accuracy, with computational model parameters showing even lower reliability; and Saeedpour et al. (2023) reported test-retest reliability of 0.40 for descriptive estimates of Pavlovian bias and 0.25 for model-based estimates over a two-week interval.

There are multiple strategies for improving the reliability of cognitive task measures (Zorowitz & Niv, 2023). For example, prior research has found that gamification, or the incorporation of (video) game design elements into cognitive tasks, can promote participant engagement (Sailer et al., 2017) and improve the reliability of task measures (Kucina et al., 2023; Verdejo-Garcia et al., 2021). Moreover, hierarchical Bayesian models – which exert a pooling effect on person-level variables, in effect correcting them for measurement error (Haines, Sullivan-Toole & Olino, 2023; Rouder & Haaf, 2019) – have been frequently shown to improve the reliability of task measures (Brown et al., 2020; Sullivan-Toole et al., 2022; Waltmann, Schlagenhauf & Deserno, 2022). Finally, practice effects can be lessened by designing tasks in such a way that prevents participants from discovering and using task-specific knowledge to enhance their performance on subsequent attempts (McLean, Mattiske & Balzan, 2018).

Here we investigate the reliability and repeatability of a novel version of the Pavlovian go/no-go task, with the aim of designing a variant of the task that is optimized for use in computational psychiatry and other individual differences research. We conducted two experiments involving two independent samples of adult participants who completed a gamified version of the task multiple times over several weeks. We used hierarchical Bayesian models to derive reinforcement-learning model-based indices of their task performance, and additionally to estimate the reliability of these measures. In Experiment 1, using a gamified version of the classic task, participants exhibited large practice effects, which negatively impacted the test-retest reliability of the performance measures. To address this issue, in Experiment 2, participants completed a modified version of the task that reduced practice effects, and led to significant improvements in the test-retest reliability of the reinforcement learning model parameters.

## Experiment 1

### Methods

#### Participants

A total of N = 148 participants were recruited in May, 2020, from Amazon Mechanical Turk via CloudResearch (Litman, Robinson & Abberbock, 2017). Participants were eligible to participate if they were at least 18 years old and resided in the United States. Following best practice recommendations (Robinson et al., 2019), no other inclusion criteria were applied. The study was approved by the Institutional Review Board of Princeton University and all participants provided informed consent. Total study duration was 15–20 minutes. Participants received monetary compensation for their time (rate: USD $12/hr), plus an incentive-compatible bonus up to $1.50 based on task performance.

Data from N = 45 participants who completed the first session were excluded prior to analysis (see “Exclusion criteria” below), leaving a final sample of N = 103 participants. These participants were re-invited to complete follow-up experiments 3, 14, and 28 days later. Once invited, participants were permitted 48 hours to complete each follow-up experiment. Retention was high for each follow-up session (Day 3: N = 94 [91.3%]; Day 14: N = 92 [89.3%]; Day 28: N = 89 [86.4%]). In addition to the performance bonus, participants received a retention bonus of $1.00 for each completed follow-up session. Detailed demographic information is presented in Table S1. The majority of participants identified as men (55 men; 47 women; 1 non-binary) and participants were 35.5 years old on average (SD = 10.3, range: 20–69 years).

#### Experimental protocol

In each session, after providing consent, participants started by completing some or all of the following self-report questionnaires: the 7-item generalized anxiety disorder scale (GAD-7; Spitzer et al., 2006); the 14-item manic and depressive tendencies scale (7-up/7-down; Youngstrom et al., 2013); and the abbreviated 12-item behavioral activation/inhibition scale (BIS/BAS; Pagliaccio et al., 2016). Participants also indicated their current mood using an affective slider (Betella & Verschure, 2016). Note that participants completed the GAD-7 and mood slider on each session, but the 7-up/7-down and BIS/BAS scales only twice (on Days 0 and 28). These measures were included for exploratory analyses not reported here.

**Figure 1:**
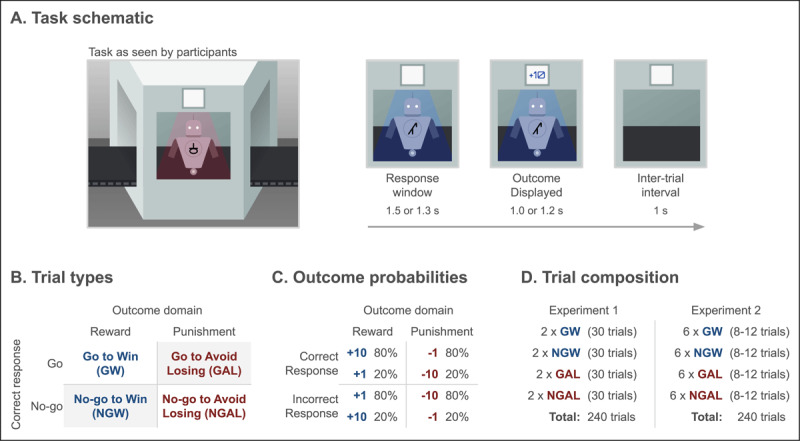
**(A) Schematic of the Pavlovian go/no-go task.** On each trial, a robot entered the ‘scanner’ from the left of screen, prompting a response (go or no-go) from the participant during a response window (Experiment 1: 1.5 seconds; Experiment 2: 1.3 seconds). The outcome (number of points won or lost) was subsequently presented on the scanner display (Experiment 1: 1.0 seconds; Experiment 2: 1.2 seconds), followed by an inter-trial interval animation (1 second) in which the conveyor belt carried the old robot out of view and a new robot into the scanner. The color of the scanner light denoted outcome domain (e.g., blue denoting reward and red denoting punishment). **(B) The four trial types**, produced by a factorial combination of outcome domain (rewarding, punishing) and correct action (go, no-go). **(C) Outcome probabilities** for each outcome domain following a correct or incorrect response. Correct responses yielded the better of the two possible outcomes with 80% chance. **(D) Trial composition.** In Experiment 1, participants saw 8 total robots (two of each trial type), each presented for 30 trials (240 total trials). In Experiment 2, participants saw 24 total robots (6 of each trial type), each for 8, 10, or 12 trials (240 total trials).

Next, participants completed a gamified version of the Pavlovian go/no-go task. In the task, participants observed different ‘robot’ stimuli (Figure 1A). On every trial, a robot was shown traveling down a conveyor belt into a ‘scanner’. Once inside, participants had 1.5 seconds to decide to either ‘repair’ the robot by pressing the space bar (“Go” response) or press nothing (“No-Go” response). A trial where there was no response within this time window was treated as a “No-Go” response, such that there were no “missed trials” and all 240 trials per participant contributed to the analyses. Participants were told that they would see different types of robots (indicated by a symbol on the robots’ chestplates), and that their goal was to learn which types of robots needed repairing based on feedback (points won/lost) following their actions.

The task involved four trial types that differed by their correct action (Go, No-Go) and outcome domain (reward, punishment; Figure 1B). Specifically, the four trial types were: go to win points (GW); no-go to win points (NGW); go to avoid losing points (GAL); and no-go to avoid losing points (NGAL). Note that GW and NGAL trials are Pavlovian-instrumental ‘congruent’ because there is a match between the correct response and the expected approach/avoidance bias due to winning or losing points for each. In contrast, NGW and GAL trials are Pavlovian-instrumental ‘incongruent’. In rewarding trials (GW, NGW), the possible outcomes were +10 or +1 points where a correct action was rewarded with +10 on 80% of the trials and +1 otherwise; in turn, an incorrect action was rewarded with +1 on 80% of the trials and +10 otherwise. In punishing trials (GAL, NGAL), outcomes were –1 or –10 points, where the correct action led to –1 on 80% of trials and the incorrect action led to –10 on 80% of trials (Figure 1C). We refer to the 80% of trials where participants received the prescribed reward magnitude for their response as having ‘veridical feedback,’ whereas the remaining 20% of trials are considered to have ‘sham feedback,’ as participants received misleading reward magnitudes that suggested a correct response was incorrect and vice versa (e.g., a correct response in the reward domain leading to +1 point). The outcome domain of each robot was explicitly signaled to participants by a blue or orange ‘scanner light’ (one color signaling reward domain and the other punishment domain, randomized within participants across sessions).

Participants saw eight unique robots in each session of the task. Each individual robot was presented for 30 trials (240 trials total; Figure 1D). Trials were divided into two blocks with four robots per block (one of each trial type). Prior to task start, participants were required to review instructions, correctly answer five comprehension questions that touched on all essential parts of the instructions, and complete several practice trials. Failing to correctly answer all comprehension questions forced the participant to reread sections of the instructions. Participants were required to complete the instructions and comprehension questions in each session. Participants were provided a break between blocks. After completing the task, participants appraised the task along three dimensions: difficulty, fun, and clarity of instructions (see Table S2). The task was programmed in jsPsych (De Leeuw, 2015) and distributed using custom web-application software (see Code Availability).

#### Exclusion criteria

To ensure data quality, data from multiple participants from the initial session were excluded prior to analysis for one or both of the following reasons: failing more than one attention check embedded in the self-report measures (i.e., incorrect response on items that resembled other items in that instrument but had obvious correct answers, such as “I was able to remember my own name”) and/or demonstrating careless responding patterns such as zigzag or straight-line responses (Kim et al., 2018; Zorowitz et al., 2023) (N = 13), or exhibiting chance-level performance (<55% correct responses) on go-to-win trials (N = 43). In total, data from N = 45 participants who completed the first session were excluded based on these criteria, leaving a final sample of N = 103 participants. No exclusions were applied to subsequent session data.

#### Descriptive analyses

We first evaluated participants’ choice behavior using five performance measures: overall percent correct responses; go bias, calculated as the difference in correct responses between Go and No-Go trials; valence bias, calculated as the difference in correct responses between rewarding and punishing trials; Pavlovian bias, which was the difference in correct responses between Pavlovian-instrumental congruent and incongruent trials; and feedback sensitivity, calculated as the difference in correct responses between trials following veridical or sham feedback (that is, following 80% of the trials where feedback aligned with the correctness of the response, and the 20% of trials with feedback matching the alternative response, respectively). Consistent with previous research (Guitart-Masip et al., 2012; Saeedpour et al., 2023), only small or nonsignificant valence biases were observed. As such, these statistics are reported only in the Supplementary Materials (Table S4).

For each session and measure, we tested if the median value across participants was significantly different than zero (or 50% for overall percent correct responses). We used the median due to skew in the performance measures. We also tested if the median value of each measure was significantly different between each pair of sessions. P-values were derived via permutation testing, where a null distribution of values was obtained by permuting the condition labels (for within-session tests) or session labels (for between-session tests) 5,000 times. Within-session tests were not corrected for multiple comparisons as each test constituted an individual hypothesis test; however, between-session tests were corrected using the family-wise error rate correction (Winkler et al., 2014) because they constituted a disjunctive test (Rubin, 2021).

#### Reinforcement learning models

To more precisely characterize participants’ performance on the Pavlovian go/no-go task, we fit a nested set of reinforcement learning models to the choice data. All models were variants of the Rescorla-Wagner model and have previously been used to predict choice behavior on this task (Guitart-Masip et al., 2012; Mkrtchian et al., 2017; Moutoussis et al., 2018; Swart et al., 2017). Under the most complex model (M7), the probability that a participant makes a go response following stimulus *k* was defined as:


1 \[
p(y=\textrm{go})=(1-\xi)\cdot\textrm{logit}^{-1}\left(\beta_{v_{k}}\cdot[Q_{k}(\textrm{Go})-Q_{k}(\textrm{NoGo})]+\tau_{v_{k}}\right)+\frac{\xi}{2}
\]

where \[
\beta_{v_{k}}
\] was the reward sensitivity (if the valence *v* of stimulus *k* was rewarding) or the punishment sensitiivty (if stimulus *k* was punishing), *Q*_*k*_(go) and *Q_k_*(no-go) were learned stimulus-action values for the go and no-go responses for stimulus *k*, respectively, \[
\tau_{v_{k}}
\] was an approach bias (if stimulus *k* was rewarding) or avoidance bias parameter (if stimulus *k* was punishing), and *ξ* was the lapse rate (i.e., the rate of choosing actions randomly due to lapse of attention). The *Q* values were learned through feedback according to a learning rule:


2 \[
Q_{k}(\textrm{action})\leftarrow\eta_{v_{k}}\cdot\left[r-Q_{k}(\textrm{action})\right]
\]

where *r* was the observed outcome on this trial and \[
\eta_{v_{k}}
\] was the learning rate or step-size parameter (*η*_+_ if stimulus k was a reward/gain domain robot, *η*_–_ if it was a punishment/loss domain robot). To allow comparison of model parameters to previous studies, and since point values are arbitrary, in our models we encoded rewards as *r* = 1 for the better of the two possible outcomes and *r* = 0 for the worse of the two possible outcomes. This was done for convenience only, and the same results are obtained when using the true point values as *r* as the two encodings are mathematically equivalent. This is because Q-values are learned separately for reward and punishment domains (as in Guitart-Masip et al., 2012) and the softmax choice function is invariant to additive constants, thus action probabilities derived from Q-values of, say, –1 and 0 are identical to those derived from Q-values of 0 and 1. As the possible reward magnitudes were instructed and the reward/punishment domain signaled on every trial, only the relative reward within condition was germane to action selection, and we therefore initialized Q-values to 0.5.

Simplifications of this model involved either fixing parameters to be equal to zero (e.g., no lapse rate) or fixing parameters to be equal for reward and punishment domains. Specifically, the base model (M1) had only two free parameters: a single outcome sensitivity parameter and a single learning rate, both shared across outcome domains (i.e., *β*_+_ = *β*_–_; *η*_+_ = *η*_–_; \[
\tau_{+}=\tau_{-}=0
\] , *ξ* = 0). Model 2 added a static action bias parameter that was shared across outcome domains (i.e., *τ*_+_ = *τ*_–_). Model 3 added to M2 independent approach (*τ*_+_) and avoidance (*τ*_–_) parameters. Models 4 and 5 respectively added to M3 independent outcome sensitivity (*β*_+_,*β*_–_; M4) or learning rate (*η*_+_,*η*_–_; M5) parameters by outcome domain. Model 6 included both independent outcome sensitivity and learning rate parameters. Finally, Model 7, the most complex model, added to M6 a potentially non-zero lapse rate (*ξ*).

All models were estimated within a hierarchical Bayesian modeling framework using Hamiltonian Monte Carlo sampling as implemented in Stan (v2.30; Carpenter et al., 2017). The hierarchical structure decomposes each parameter into group mean, participant-specific, and session-specific components (see Equation 3, below). This decomposition allows the model to separate stable individual differences from session-to-session variability and measurement noise, with the pooling effect occurring because individual estimates are informed by both that person’s data and group-level patterns.

For each model, four separate chains with randomized start values each drew 7,500 samples from the posterior. Each chain generated 5,000 warm-up samples and 2,500 post-warmup samples. The warm-up samples were discarded, and every even numbered sample of the remaining samples was discarded via thinning (thin = 2), retaining 1,250 post-warmup samples per chain for a total of 5,000 samples overall for parameter estimation (1,250 × 4 chains). The \[
\hat{R}
\] values for all parameters were ≤ 1.01, indicating acceptable convergence between chains, and there were no divergent transitions in any chain. For all models, we specified priors that reflected reasonable assumptions about parameter ranges and distributions based on the task design and participant selection criteria (Table S3). The learning-rate priors assumed a weak bimodal distribution reflecting expected heterogeneity, while the lapse-rate prior concentrated mass below 0.5 given our quality-control procedures that excluded participants with chance or below-chance performance.

Fits of the models to behavioral data were assessed using posterior predictive checks. Specifically, we inspected each model’s ability to reproduce both group-averaged learning curves by trial type and each participant’s proportion of go responses by trial type. Model fits were compared using approximate leave-one-trial-out cross-validation via Pareto smoothed importance sampling (PSIS-LOO; Vehtari, Gelman & Gabry, 2017). (Note this may, in principle, differ from cross-validation at the participant level, which has been argued to be a relevant unit of exchangeability at which to compare models (Stephan et al., 2009).) We considered a difference in PSIS-LOO values that is four times larger than the mean PSIS-LOO standard error as a significant improvement in model fit due to additional parameters (Vehtari, 2023).

We also investigated the reliability of the model parameters for the best-fitting model using a Bayesian hierarchical modeling framework, in which data were pooled within and across participants (Rouder & Haaf, 2019). After identifying the best-fitting model architecture using approximate LOO cross-validation, we re-estimated this model with session-specific (for test-retest reliability) or block-specific (for split-half reliability) group-level parameters while maintaining the hierarchical structure that pools information across participants. Specifically, each parameter \[
\theta\in\{\beta_{+},\beta_{-},\eta_{+},\tau_{+},\tau_{-},\xi\}
\] was estimated as follows:


3 \[
\begin{split}\theta_{i1}=\mu_{1}+\theta_{ic}-\theta_{id}\\[3.0pt]\theta_{i2}=\mu_{2}+\theta_{ic}+\theta_{id}\end{split}
\]

where *θ*_*i*1_ and *θ*_*i*2_ are a given parameter (e.g., reward sensitivity, *β*_+_) for participant *i* in sessions or blocks 1 and 2, respectively; *μ*_1_ and *μ*_2_ are the group-averaged parameters for sessions or blocks 1 and 2 estimated jointly with individual-level parameters; *θ*_*ic*_ is the common effect for participant *i* (i.e., the component of a participant-level parameter that is different from the group mean and stable across sessions or blocks); and *θ*_*id*_ is the difference effect for participant *i* (i.e., the parameter component that is variable across sessions or blocks). The collection of *θ*_*ic*_ parameters constituted between-participants variability, whereas the collection of *θ*_*id*_ parameters constituted within-participants variability. Both *θ*_*ic*_ and *θ*_*id*_ were assumed to be normally distributed with zero means and independent estimated variances. Split-half and test-retest reliability estimates were calculated by taking both Spearman correlations and intraclass correlation (ICC) coefficients of *θ*_*i*1_ and *θ*_*i*2_ across task blocks and sessions, respectively (Brown et al., 2020; Pike et al., 2022). We used the Spearman correlation because we were primarily interested in the consistency of rank ordering of participants’ parameter estimates over time. We calculated ICC as the ratio of between-participant variance to total variance ( \[
ICC=\frac{\sigma^{2}_{between}}{(\sigma^{2}_{between}+\sigma^{2}_{within})}
\] ), which provides a measure of the proportion of total variance attributable to stable individual differences. Although arbitrary, we followed convention and defined *ρ* ≥ 0.7 and *r*_ICC_ ≥ .6 as the thresholds for “acceptable”, and “good” reliability, respectively (Cicchetti, 1994).

### Results

#### Descriptive analyses

**Figure 2:**
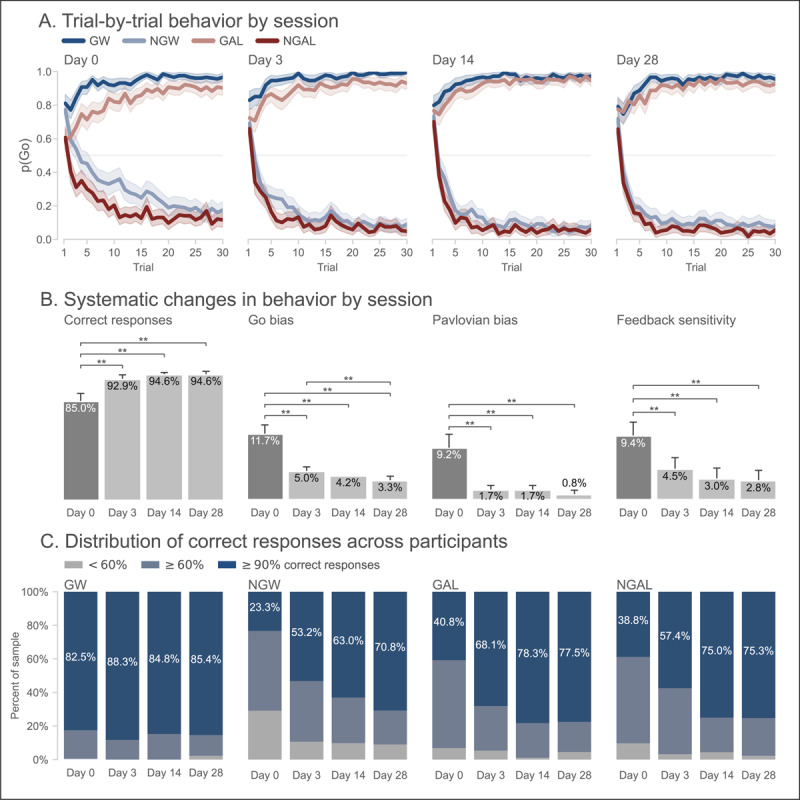
**Large practice effects on the standard Pavlovian go/no-go task in Experiment 1. (A) Group-averaged learning curves** for each trial type and session. Shaded regions indicate 95% bootstrapped confidence intervals. **(B) Group-averaged performance for each session.** Performance measures from left-to-right: Correct responses, or overall accuracy; Go bias, or difference in accuracy between Go and No-Go trials; Congruence effect, or difference in accuracy between congruent (GW, NGAL) and incongruent (NGW, GAL) trials; and Feedback sensitivity, or the difference in accuracy on trials following veridical and sham feedback. Behavior on the first session was significantly different from all other sessions on all measures. ** Denotes significant pairwise difference (*p*<0.05, corrected for multiple comparisons). **(C) Distribution of correct responses across sessions by trial type.** Percentage of participants, for each session and trial type, exhibiting at- or below-chance performance ( \[
<60\%
\] response accuracy; grey), intermediate performance ( \[
\geq 60\%
\] response accuracy; light blue), or near-perfect performance ( \[
\geq 90\%
\] response accuracy; dark blue). Across sessions, performance improved on all trial types that were not already close to ceiling on the first session.

Trial-by-trial choice behavior for each session is presented in Figure 2A. Performance in the first session qualitatively conformed to the expected pattern of results (i.e., worse performance on Pavlovian-instrumental incongruent trials [GAL, NGW]). However, this effect seemed diminished in all follow-up sessions. Indeed, group-averaged performance measures by session (Figure 2B; complete descriptive statistics are reported in Table S4) showed that participants made the correct response on 85.0% of trials on the first session (Day 0), which increased to near-ceiling levels in all subsequent sessions. Pairwise comparisons confirmed that performance was indeed worse on Day 0 compared to each follow-up session (all *p*<0.001); no other comparisons were significant. Participants’ self-reported mood and anxiety were largely stable over the same period (Figure S1), indicating this shift in performance more likely reflects practice effects rather than changes in participants’ state.

Across sessions, participants made more correct responses on Go trials than on No-Go trials. However, this “Go bias” was significantly reduced in all follow-up sessions compared to Day 0 (all *p*<0.001); so too was it on Day 28 compared to Day 3 (*p*<0.001). Similarly, participants made more correct responses on congruent than incongruent trials. As with the Go bias, this “Pavlovian bias” was significantly reduced in all follow-up sessions compared to Day 0 (all *p*<0.001; no other comparisons were significant).

Feedback sensitivity also diminished from the first to later sessions. Across sessions, participants made more correct responses following veridical compared to sham feedback (all *p*<0.001). However, feedback sensitivity was significantly reduced in all follow-up sessions compared to Day 0 (all *p*<0.001; no other comparisons were significant) suggesting that feedback had less of an effect on choice in later sessions. This is consistent with participants’ learning curves which show, in all days except Day 0, that participants quickly learned the correct action for each stimulus and maintained this policy despite the 20% sham feedback (Figure 2A).

These results summarize group-averaged performance. To gain insight into individual differences, Figure 2C shows the proportion of participants who exhibited chance-level (<60% correct responses), intermediate ( ≥ 60% and <90%), or near-ceiling performance ( ≥ 90%) by session and trial type. Excepting GW trials, where performance of over 80% of participants was close to ceiling already in the first session, the percentage of participants nearing ceiling-level performance increases from a minority on Day 0 to the majority of participants in all follow-up sessions. Two-way chi-squared tests confirmed this trend (GW: *χ*^2^(6) = 8.149, *p* = 0.227; NGW: *χ*^2^(6) = 55.458, *p*<0.001; GAL: *χ*^2^(6) = 42.191, *p*<0.001; NGAL: *χ*^2^(6) = 39.287, *p*<0.001). In sum, the improvements in task performance (and accompanying reductions in choice biases) with repeat testing observed at the group-level extended to the majority of participants.

#### Model comparison

The results of the model comparison are summarized in Table 1. Collapsing across sessions, the best-fitting model was the most complex one (i.e., the model including independent reward sensitivity, learning rate and approach/avoidance bias parameters per outcome domain, plus a lapse rate; M7). Importantly, this was also the best-fitting model within each session (Table S5). Posterior predictive checks indicated that this model provided excellent fits to the choice data from each session (Figure S3).

**Table 1:** **Model comparison collapsing across sessions.** Accuracy = trial-level choice prediction accuracy between observed and model-predicted Go responses. PSIS-LOO = approximate leave-one-out cross-validation scores presented in deviance scale (smaller numbers indicate better fit). ΔPSIS-LOO = difference in PSIS-LOO values between each model and the best-fitting model (M7).


MODEL	PARAMETERS	ACCURACY	PSIS-LOO	\[ \symbf\Delta \] PSIS-LOO (se)

M1	*β,η*	87.5%	–151457.9	–5602.6 (68.3)

M2	\[ \beta,\tau,\eta \]	89.0%	–154011.9	–3048.6 (51.2)

M3	\[ \beta,\tau_{+},\tau_{-},\eta \]	89.8%	–155817.8	–1242.7 (31.3)

M4	\[ \beta_{+},\beta_{-},\tau_{+},\tau_{-},\eta \]	89.8%	–156261.6	–798.8 (22.6)

M5	\[ \beta,\tau_{+},\tau_{-},\eta_{+},\eta_{-} \]	89.9%	–156265.9	–794.6 (20.7)

M6	\[ \beta_{+},\beta_{-}\tau_{+},\tau_{-},\eta_{+},\eta_{-} \]	89.9%	–156401.8	–658.6 (18.8)

M7	\[ \beta_{+},\beta_{-}\tau_{+},\tau_{-},\eta_{+},\eta_{-},\xi \]	90.1%	–157060.5	–


#### Model parameters

**Figure 3:**
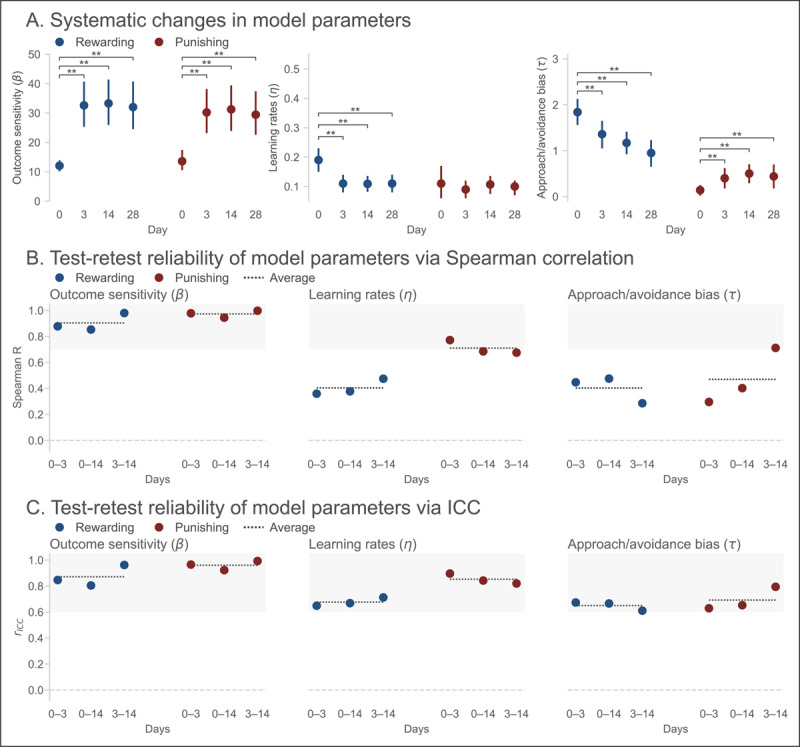
**Reinforcement learning model parameters in Experiment 1 show evidence of practice effects and low reliability. (A)** Group-level model parameters for each session. Error bars indicate 95% Bayesian confidence intervals (CIs). ** Denotes pairwise comparison where 95% CI of the difference excludes zero. **(B)** Test-retest reliability estimates for each model parameter. Dotted lines indicate average across pairs of sessions. Shaded region indicates conventional range of acceptable reliability (*ρ* ≥ 0.7). **(C)** Test-retest reliability estimates for each model parameter using ICC. Dotted lines indicate average across the three sessions. Shaded region indicates conventional range of good reliability (*r*_ICC_ ≥ 0.6).

Figure 3A shows the estimated group-level parameters from the best-fitting model. Consistent with the descriptive analyses above, large shifts in parameter values were observed following Day 0. The reward and punishment sensitivity parameters (*β*_+_, *β*_–_) exhibited an almost threefold increase between Days 0 and 3, and stabilized thereafter. The inverse pattern was observed for the reward learning rate (*η*_+_). Crucially, the approach/avoidance bias parameters followed a similar pattern. The approach bias (*τ*_+_) decreased significantly between Days 0 and 3, and qualitatively declined thereafter. In turn, the avoidance bias (*τ*_–_) increased significantly between Days 0 and 3, and stabilized thereafter. That is, Pavlovian biases diminished in absolute and relative terms (i.e., compared to the outcome sensitivity parameters) with repeat testing.

The test-retest reliability estimates for each model parameter are presented in Figure 3B–C. The results were mixed. Averaging across session pairs, acceptable test-retest reliability was observed for the outcome sensitivity parameters (*β*_+_: *ρ* = 0.903, 95% CI = [0.873, 0.919], *r*_ICC_ = 0.871, 95% CI = [0.806, 0.956]; *β*_–_: *ρ* = 0.973, 95% CI = [0.959, 0.979], *r*_ICC_ = 0.960, 95% CI = [0.920, 0.991]) and the punishment learning rate (*η*_–_: *ρ* = 0.711, 95% CI = [0.633, 0.771], *r*_ICC_ = 0.853, 95% CI = [0.821, 0.893]).

Conversely, test-retest reliability was unacceptable according to Spearman correlation coefficients for the approach and avoidance bias parameters (*τ*_+_: *ρ* = 0.402, 95% CI = [0.290, 0.501]; *τ*_–_: *ρ* = 0.470, 95% CI = [0.369, 0.554]) and the reward learning rate (*η*_+_: *ρ* = 0.403, 95% CI = [0.294, 0.497]), though ICC estimates for these parameters reached “good” reliability thresholds (*τ*_+_: *r*_ICC_ = 0.650, 95% CI = [0.613, 0.673]; *τ*_–_: *r*_ICC_ = 0.692, 95% CI = [0.630, 0.787]; *η*_+_: *r*_ICC_ = 0.676, 95% CI = [0.649, 0.710]). A similarly mixed pattern was observed for the split-half reliability estimates (Figure S5A).

### Discussion

Our goal was to evaluate the stability and reliability of individual differences in performance on a gamified version of the popular Pavlovian go/no-go task. At both the group and participant levels, we observed significant practice effects following the first session. An increasing majority of participants exhibited near-ceiling performance, across trial types, with each additional task administration. Consequently, the magnitude of group-averaged behavioral effects including the go bias, Pavlovian bias, and feedback sensitivity were diminished by half or more after the first session. This was reflected in the group-level parameters of a reinforcement learning model fit to participants’ choice data, which indicated that Pavlovian biases were significantly attenuated in follow-up sessions. Consequently, we found that the Pavlovian bias parameters exhibited poor-to-moderate test-retest reliability. This last result is perhaps unsurprising insofar that low between-participants variability diminishes reliability (Zorowitz & Niv, 2023).

The results of Experiment 1 raise two questions: what underlies these practice effects and what can be done to mitigate or prevent them? With respect to the first question, one possibility is that, after the initial session, participants rely on the already learned structure of the task to solve it more effectively. Specifically, in the canonical Pavlovian go/no-go task, for every Go stimulus (e.g., GW) there is a corresponding No-Go stimulus (e.g., NGW). As such, learning the correct action for one stimulus provides information about the correct action for its complement. Recognizing this, savvy participants may forego reinforcement learning in favor of a process-of-elimination strategy to deduce which is the Go and which is the No-Go stimulus in each pair. Indeed, feedback from several participants in this study suggested that they may have utilized this form of top-down strategy. This interpretation is further supported by reaction time data (Figure S6), which showed that participants in Experiment 1 became significantly faster across sessions, suggesting increasingly automatic responding consistent with learned task structure. In contrast, Experiment 2 showed stable reaction times across sessions, aligning with the reduced practice effects observed in that version.

This suggests that a version of the task with a less predictable trial structure might reduce practice effects. By eliminating the dependence between stimuli, motivated participants aiming to maximize their performance should have no strategy better than learning from the feedback for each of their actions. By minimizing practice effects and increasing between-participants variability, it is plausible that parameter reliability would also improve. In the next experiment, we investigated precisely this.

## Experiment 2

### Methods

#### Participants

A total of N = 156 participants were recruited in December, 2020, from Amazon Mechanical Turk via CloudResearch (Litman, Robinson & Abberbock, 2017). Inclusion criteria were the same as in Experiment 1. The study was approved by the Institutional Review Board of Princeton University, and all participants provided informed consent. Total study duration was again 15-20 minutes. Monetary compensation, including the performance bonus, was the same as in Experiment 1.

Data from N = 46 participants who completed the first session were excluded prior to analysis (see “Exclusion criteria” below), leaving a final sample of N = 110 participants. These participants were re-invited to complete follow-up experiments 3 and 14 days later. (There was no follow-up session at 28 days due to overlap with the Christmas holiday.) Once invited, participants were permitted 48 hours to complete the follow-up experiment. Participant retention was again high for each follow-up session (Day 3: N = 97 [88.2%]; Day 14: N = 99 [90.0%]). Participants again received a retention bonus of $1.00 for each completed follow-up session. Detailed demographic information is presented in Table S1. The majority of participants identified as men (65 men; 53 women; 1 non-binary individual; 1 rather not say) and were 39.6 years old on average (SD = 11.52, range: 23–69 years).

#### Experimental protocol

The overall experimental protocol for Experiment 2 was almost identical to Experiment 1. In each session, participants started by completing the same self-report questionnaires with the exception that the 7-up/7-down was replaced with the 7-item depression subscale from the depression, anxiety, and stress scale (DASS; Henry & Crawford, 2005). Participants completed the BIS/BAS scale once (on Day 0), but completed the GAD-7, DASS, and mood slider scales at the start of every session. These measures were included for exploratory analyses not reported here.

Next, participants completed a modified version of the gamified Pavlovian go/no-go task with a trial structure similar to (Wittmann et al., 2008). In particular, instead of 8 unique robots each presented for 30 trials, participants saw a total of 24 unique robots presented for 8, 10, or 12 trials each. Each robot was presented for fewer trials as we were interested in measuring the learning process, where the expression of Pavlovian biases is typically largest, rather than asymptotic performance. Robots were presented to participants in mini-batches, each involving four robots and totaling approximately 40 trials. Crucially batches were not required to represent all four trial types (see Figure S2 for an example). That is, in any section of the task, participants were not guaranteed to observe one of each type of robot. As such, learning about one robot did not imply information about another robot and participants could not rely on a top-down process-of-elimination strategy. Participants completed six mini-batches, which were divided into two blocks of 120 trials each (12 unique robots per block; three robots of each trial type; Figure 1D).

The task was visually similar to Experiment 1 except in two respects. First, the scanner colors were now blue and red (instead of blue and orange), and fixed such that blue always indicated rewarding trials and red indicated punishing trials. This was intended to align better with natural reward and punishment domains and potentially enhance Pavlovian biases (Elliot & Maier, 2012; Mehta & Zhu, 2009; Xia et al., 2016). This design choice represents a departure from the Guitart-Masip paradigm (Guitart-Masip et al., 2012), where outcome domains were not signaled. We chose to signal the outcome domain explicitly, and do so in alignment with learned color mappings, with the goal of enhancing the Pavlovian biases we were attempting to measure and to avoid a period in which participants need to learn the mapping between each robot type and the relevant outcome domain (that was especially important given that our design presented each robot for few trials). Second, the symbols on the robots’ chestplates were drawn from one of two Brussels Artificial Character Sets (Vidal, Content & Chetail, 2017) or the English alphabet (randomized within participants across sessions). These new symbols were used in order to accommodate the need for three times the number of distinctly recognizable robots. Pairwise comparisons revealed no significant differences in percent correct responses by character set (all *p*>0.90, corrected for multiple comparisons). The timing of the task was also unchanged except the response window was shortened (from 1.5 to 1.3 seconds) and the feedback window was lengthened (from 1.0 to 1.2 seconds).

#### Exclusion criteria

Data from N = 46 participants who completed the experiment on Day 0 were excluded prior to analysis for one or more of the following reasons: failing one or more attention checks embedded in the self-report measures (providing an incorrect response on items with obvious correct answers and/or showing careless responding patterns such as zigzag or straight-line responses Kim et al. (2018) and Zorowitz et al. (2023); N = 30), making either all Go or all No-Go responses on more than 90% of trials (N = 5), or exhibiting chance-level performance on go-to-win trials ( \[
{<}55\%
\] correct responses; N = 22). These exclusion criteria left a final sample of N = 110 participants. No exclusions were applied to subsequent session data.

#### Analyses

Analyses for Experiment 2 were identical to those for Experiment 1. The only exception was the sampling procedure: each chain drew 6,250 samples from the posterior (5,000 warm-up samples and 1,250 post-warmup samples) with no thinning applied (thin = 1), which yielded an equivalent total of 5,000 post-warmup samples for parameter estimation (1,250 × 4 chains). In addition, we performed Wald tests to compare the magnitude of choice and practice effects between Experiments 1 and 2. P-values were derived from permutation testing, where a null distribution of values was obtained by permuting the experiment (1 or 2) and session labels (1, 2, or 3), across and within participants, respectively, 5,000 times.

### Results

#### Descriptive analyses

**Figure 4:**
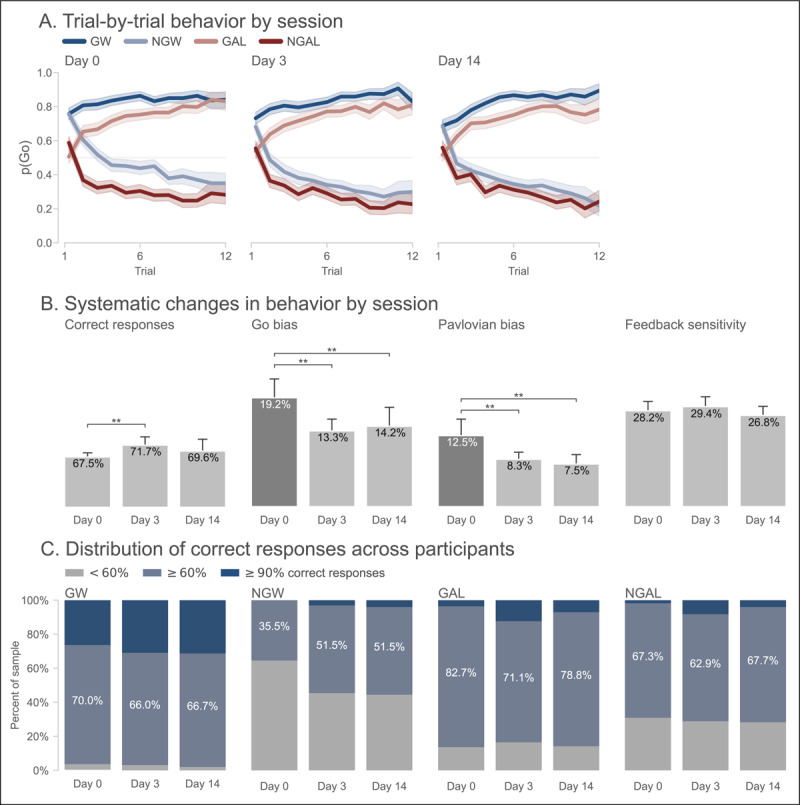
**Smaller or no practice effects on the modified Pavlovian go/no-go task in Experiment 2. (A)** Group-averaged learning curves for each trial type and session. Shaded regions indicate 95% bootstrapped confidence intervals. **(B)** Group-averaged performance for each session. Performance indices from left-to-right: Correct responses, or overall accuracy; Go bias, or difference in accuracy between Go and No-Go trials; Congruency effect, or difference in accuracy between Pavlovian congruent (GW, NGAL) and incongruent (NGW, GAL) trials; and Feedback sensitivity, or the difference in accuracy on trials following veridical and sham feedback. ** Denotes significant pairwise difference (*p*<0.05, corrected for multiple comparisons). **(C)** The percentage of participants, for each session and trial type, exhibiting at- or below-chance performance ( \[
{<}60\%
\] response accuracy; grey), intermediate performance ( \[
{\geq}60\%
\] and \[
{<}90\%
\] response accuracy; light blue), or near-perfect performance ( \[
{\geq}90\%
\] response accuracy; dark blue).

Figure 4A shows trial-by-trial choice behavior for each session of the experiment. In contrast to Experiment 1 (c.f. Figure 2A), performance in all sessions conformed to the expected pattern of results. Group-averaged performance measures per session (Figure 4B) show that while performance improved after Day 0, improvement was only marginal. In particular, pairwise comparisons showed performance was significantly better on Day 3 compared to Day 0 (*p* = 0.009); however, no other pairwise comparisons were significant (complete descriptive statistics are reported in Table S6). In comparison to Experiment 1, performance accuracy on the modified task was lower (mean difference = 21.2%; F(1,589) = 518.618, *p*<0.001). This is to be expected given that the modified task was designed in part to prevent participants from reaching asymptotic performance. Crucially, practice effects (defined as the average difference in performance between the first and all follow-up sessions) were significantly reduced for the modified task in comparison to Experiment 1 (mean difference = –5.6%; F(1,589) = 8.373, *p*<0.001).

In all sessions, participants performed better on Go trials than on No-Go trials (“Go bias”). The Go bias on Day 0 was significantly greater than that for all other sessions (all *p*<0.005); no other between-session comparisons were significant. And although the practice effect for the Go bias was numerically smaller for the modified task, it was not significantly different than that for Experiment 1 (mean difference = –1.7%; F(1,589) = 0.760, *p* = 0.388). Nevertheless, Go biases across sessions were significantly greater than those observed in Experiment 1 (mean difference = 8.6%; F(1,589) = 88.026, *p*<0.001).

Participants also performed better on Pavlovian-instrumental congruent compared to incongruent trials in all sessions, manifesting a Pavlovian bias. The Pavlovian bias on Day 0 was significantly greater than that for all other sessions (both *p* = 0.027); no other between-session comparisons were significant. Unlike the Go bias, the practice effect for the Pavlovian bias was significantly reduced for the modified task in comparison to Experiment 1 (mean difference = –2.9%; F(1,589) = 4.173, *p* = 0.037). And like the Go bias, Pavlovian biases were significantly greater than those observed in Experiment 1 (mean difference = 5.3%; F(1,589) = 59.284, *p*<0.001).

Regarding feedback sensitivity, across sessions participants made more correct responses following veridical compared to sham feedback. No pairwise comparison between sessions was significant (all *p*>0.10), suggesting that feedback sensitivity was largely conserved across sessions. As a result, the practice effect for feedback sensitivity was significantly smaller in comparison to Experiment 1 (mean difference = –5.4%; F(1,589) = 8.591, *p*<0.001). Moreover, feedback sensitivity was significantly greater across sessions than that observed in Experiment 1 (mean difference = 22.4%; F(1,589) = 643.245, *p*<0.001). In sum, group-averaged behavior on the modified task showed evidence of residual practice effects. However, despite this, the expected choice biases were significantly larger than those observed in Experiment 1 and practice effects on the modified task were, with one exception, significantly reduced.

Turning next to individual variation in performance, the proportion of participants who exhibited chance-level, intermediate, or near-ceiling performance by session and trial type is presented in Figure 4C. In contrast to Experiment 1, ceiling performance was relatively rare and the majority of participants exhibited intermediate levels of performance across all trial types and sessions (the only exception was for NGW trials on Day 0, where the majority of participants showed chance-level performance). Two-way chi-squared tests of independence confirmed that, with an exception for NGW trials, no significant shift in participants’ performance across sessions was observed (GW: *χ*^2^(4) = 1.163, *p* = 0.884; NGW: *χ*^2^(4) = 13.343, *p* = 0.010; GAL: *χ*^2^(4) = 6.499, *p* = 0.165; NGAL: *χ*^2^(4) = 5.097, *p* = 0.278). Thus, the majority of participants exhibited and maintained intermediate levels of performance on the modified Pavlovian go/no-go task.

#### Model comparison

Results of the model comparison are summarized in Table 2. Trial-level choice prediction for all models was worse in Experiment 2 than in Experiment 1, which is to be expected insofar as it is easier to predict asymptotic behavior, whereas the modified task primarily measures participants’ performance during learning (i.e., when choice is most stochastic). As in Experiment 1, collapsing across sessions, the best-fitting model was M7, the most complex model. This was also the best-fitting model within each session (Table S7). Posterior predictive checks indicated that this model provided excellent fits to the choice data from each session (Figure S4).

**Table 2:** **Model comparison collapsing across sessions.** Accuracy = trial-level choice prediction accuracy between observed and model-predicted Go responses. PSIS-LOO = approximate leave-one-out cross-validation presented in deviance scale (smaller numbers indicate better fit). ΔPSIS-LOO = difference in PSIS-LOO values between each model and the best-fitting model (M7).


MODEL	PARAMETERS	ACCURACY	PSIS-LOO	\[ \symbf\Delta \] PSIS-LOO (se)

M1	*β,η*	72.9%	–95806.3	–6205.2 (73.2)

M2	\[ \beta,\tau,\eta \]	76.5%	–99616.0	–2395.5 (48.9)

M3	\[ \beta,\tau_{+},\tau_{-},\eta \]	77.6%	–101283.0	–728.5 (28.2)

M4	\[ \beta_{+},\beta_{-},\tau_{+},\tau_{-},\eta \]	77.5%	–101422.4	–589.0 (21.1)

M5	\[ \beta,\tau_{+},\tau_{-},\eta_{+},\eta_{-} \]	77.7%	–101519.0	–492.4 (19.1)

M6	\[ \beta_{+},\beta_{-}\tau_{+},\tau_{-},\eta_{+},\eta_{-} \]	77.8%	–101548.7	–462.7 (17.2)

M7	\[ \beta_{+},\beta_{-}\tau_{+},\tau_{-},\eta_{+},\eta_{-},\xi \]	78.1%	–102011.4	–


#### Model parameters

**Figure 5:**
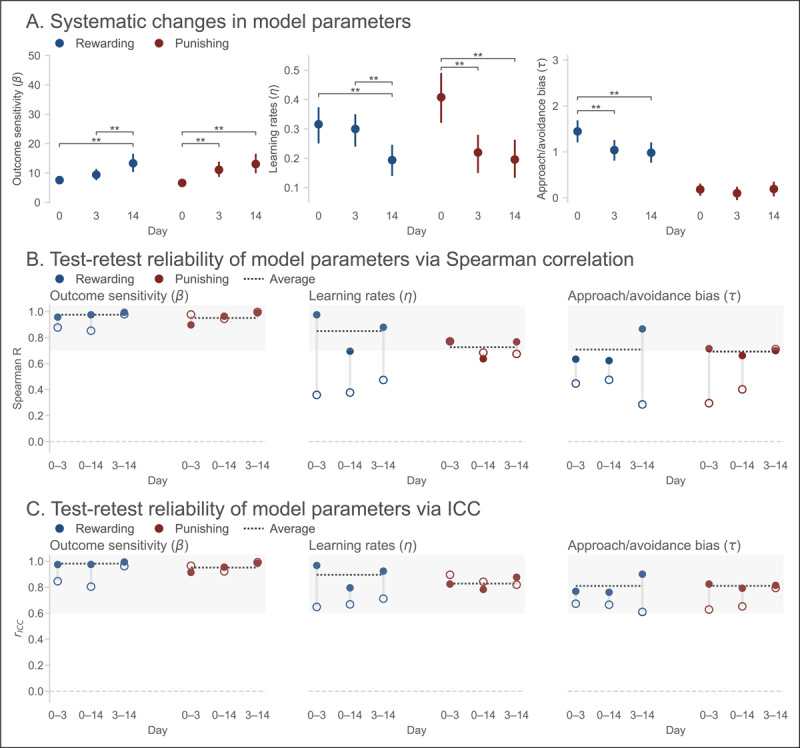
**Reinforcement learning model parameters in Experiment 2 show improved stability and reliability. (A)** Group-level model parameters for each session. Error bars indicate 95% Bayesian confidence intervals (CIs). ** Denotes pairwise comparison where 95% CI of the difference excludes zero. **(B)** Test-retest reliability estimates for each model parameter. Filled circles denote estimates for Experiment 2; open circles denote estimates from Experiment 1, for comparison. Grey vertical lines show the change in reliability across experiments. Dotted lines indicates average reliability for Experiment 2. Shaded region indicates conventional range of acceptable reliability (*ρ* ≥ 0.7). **(C)** Test-retest reliability estimates for each model parameter using ICC. Dotted lines indicate average across pairs of sessions. Shaded region indicates conventional range of good reliability (*r*_ICC_ ≥ 0.6).

The estimated group-level parameters from the best-fitting model are presented in Figure 5A. In comparison to Experiment 1, we observed smaller but still significant changes in the reward and punishment sensitivity parameters across days. Specifically, reward sensitivity (*β*_+_) was significantly larger on Day 14 compared to Days 0 and 3, whereas punishment sensitivity (*β*_–_) was significantly larger on Days 3 and 14 compared to Day 0. Both the reward and punishment sensitivity parameters were on average smaller in Experiment 2 as compared to Experiment 1 (reward sensitivity: mean difference between experiments = –15.895, 95% CI = [11.934, 19.832]; punishment sensitivity: mean difference = –14.771, 95% CI = [10.531, 18.708]). Practice effects manifest in this task as increases in the proportion of correct responses in follow-up sessions. In the model, this appears as a between-sessions increase in the reward and punishment sensitivity parameters. Therefore, one way to quantify practice effects is as the difference in reward and punishment sensitivity parameters between Day 0 and the average of all other days. This difference was significantly smaller in Experiment 2 compared to Experiment 1 (reward sensitivity: mean difference = –17.106, 95% CI = [–23.753, –11.385]; punishment sensitivity: mean difference = –11.700, 95% CI = [–18.486, –4.777]).

The inverse pattern was observed for reward (*η*_+_) and punishment learning rates (*η*_–_): reward learning rates were significantly higher on Day 14 compared to Days 0 and 3, while punishment learning rates were significantly lower on Days 3 and 14 compared to Day 0. In comparison to Experiment 1, both reward and punishment learning rates were greater on average (reward learning rate: mean difference = 0.133, 95% CI = [0.094, 0.170]; punishment learning rate: mean difference = 0.186, 95% CI = [0.079, 0.301]). Practice effects for reward learning rates were not significantly different between the two experiments (mean difference = –0.015, 95% CI = [–0.098, 0.068]), but were in fact larger for the punishment learning rate in Experiment 2 (mean difference = 0.186, 95% CI = [0.079, 0.301]).

Finally, the approach bias (*τ*_+_) was slightly but significantly larger on Day 0 compared to Days 3 and 14. No significant differences across sessions were observed in the avoidance bias (*τ*_–_). Therefore, although Pavlovian biases were somewhat diminished through repeated testing, in both absolute and relative terms (i.e., compared to the outcome sensitivity parameters), they remained largely intact in later sessions. Relative to the magnitude of the reward sensitivity parameter, the approach bias was significantly larger on average in the modified task than in Experiment 1 (mean difference = 0.049, 95% CI = [0.027, 0.071]), although practice effects between the two experiments were not significantly different (mean difference = –0.015, 95% CI = [–0.070, 0.041]). Also in relative terms, the avoidance bias was not significantly different between the two experiments (mean difference = 0.004, 95% CI = [–0.006, 0.015]), nor was the difference in practice effects (mean difference = 0.020, 95% = [–0.005, 0.045]). The Pavlovian bias (defined here as the difference between the approach and avoidance parameters) was significantly greater in the modified task compared to Experiment 1 (mean difference = 0.045, 95% CI = [0.022, 0.071]). Thus, in line with the descriptive results, Pavlovian biases were larger in the modified task despite the residual practice effects.

The estimated test-retest reliability of the model parameters is presented in Figure 5B–C. In contrast to Experiment 1, acceptable test-retest reliability was observed for essentially all parameters when averaging across session pairs. For outcome sensitivity parameters (*β*_+_: *ρ* = 0.976, 95% CI = [0.962, 0.982], *r*_ICC_ = 0.981, 95% CI = [0.975, 0.993]; *β*_–_: *ρ* = 0.951, 95% CI = [0.925, 0.964], *r*_ICC_ = 0.951, 95% CI = [0.924, 0.991]), learning rates (*η*_+_: *ρ* = 0.850, 95% CI = [0.800, 0.882], *r*_ICC_ = 0.896, 95% CI = [0.802, 0.965]; *η*_–_: *ρ* = 0.726, 95% CI = [0.655, 0.780], *r*_ICC_ = 0.852, 95% CI = [0.821, 0.893]), and approach/avoidance bias parameters (*τ*_+_: *ρ* = 0.708, 95% CI = [0.629, 0.771], *r*_ICC_ = 0.810, 95% CI = [0.761, 0.894]; *τ*_–_: *ρ* = 0.692, 95% CI = [0.617, 0.750], *r*_ICC_ = 0.692, 95% CI = [0.630, 0.787]), both correlation and ICC estimates indicated good to excellent reliability.

Compared to Experiment 1, test-retest reliability was significantly improved for reward sensitivity (change in average *ρ* = 0.072, 95% CI = [0.062, 0.088]), approach bias (change in average *ρ* = 0.305, 95% CI = [0.269, 0.708]), avoidance bias (change in average *ρ* = 0.222, 95% CI = [0.196, 0.248]), and reward learning rate (change in average *ρ* = 0.446, 95% CI = [0.385, 0.505]); no parameters showed significantly worsened reliability. A similar pattern of results was observed for the split-half reliability estimates (Figure S5B).

### Discussion

The goal of the second experiment was to evaluate the stability and reliability of individual differences in performance on a modified version of the Pavlovian go/no-go task that was designed to keep participants learning and to lessen practice effects. At the group level, participants showed the desired behavioral effects (e.g., go bias, Pavlovian bias, and feedback sensitivity) at significantly greater levels than observed in Experiment 1 across all sessions. Although participants continued to exhibit practice effects on the modified task, these were significantly reduced for the majority of task performance indices. Moreover, the fraction of participants maintaining an intermediate level of performance was largely conserved across sessions. These findings were reflected in the parameters of a reinforcement learning model fit to participants’ choice data, where parameters were largely stable and consequently exhibited acceptable test-retest reliability.

## General Discussion

Despite considerable use in individual-differences and computational psychiatry research, previous studies of the psychometric properties of the Pavlovian go/no-go task found that both descriptive and model-based measures of task performance showed poor reliability (Moutoussis et al., 2018; Pike et al., 2022; Saeedpour et al., 2023). Here, we investigated the psychometric properties of two variants of the task in an attempt to develop a more reliable version – one that would be usable in clinical practice where patients may perform a task multiple times (e.g., before, during, and after treatment). In the first experiment, we used a gamified version of the standard task. Here, we observed considerable practice effects whereby the majority of participants exhibited near-ceiling levels of performance with repeat testing. Consequently, the test-retest reliability of multiple reinforcement-learning model parameters estimated from participants’ behavior was unacceptable. To address these issues, in Experiment 2 we designed a version of the task that measures choice behavior primarily during learning and prevents undesirable process-of-elimination strategies. Participants exhibited reduced practice effects on this version of the task and, as a consequence, the test-retest reliability of reinforcement-learning model parameters was significantly improved.

The estimates of model-parameter reliability observed in both our experiments were larger than previously reported for the Pavlovian Go/No-Go task (Moutoussis et al., 2018; Pike et al., 2022; Saeedpour et al., 2023). This likely reflects a confluence of factors. First, both versions of the task studied here were gamified. Gamification has previously been shown to promote participant engagement and minimize confusion (Sailer et al., 2017) and benefit the reliability of cognitive task measures (Kucina et al., 2023; Verdejo-Garcia et al., 2021). Second, we used a hierarchical Bayesian modeling framework to estimate model parameters for the reliability analyses. Hierarchical models exert a pooling or regularization effect on model parameters, which decreases measurement error and improves estimates of reliability (Haines, Sullivan-Toole & Olino, 2023; Rouder & Haaf, 2019). Indeed, our results are consistent with previous empirical studies that have demonstrated the benefits of hierarchical Bayesian models for estimating parameter reliability (Brown et al., 2020; Waltmann, Schlagenhauf & Deserno, 2022). Finally, in Experiment 2, we redesigned the trial structure of the Pavlovian go/no-go task such as to prevent practice effects. Practice effects can harm reliability when they induce ceiling performance (as in Experiment 1) or when they are not uniformly expressed by participants (e.g., as a function of age (Anokhin et al., 2022)). It is possible that such effects worsened reliability estimates in a prior study where practice effects were observed in an adolescent sample (Moutoussis et al., 2018).

The occurrence of practice effects with repeated administrations is common for cognitive tasks (Hausknecht et al., 2007; Scharfen, Peters & Holling, 2018). Practice effects may reflect a number of factors, such as reductions in performance anxiety or the acquisition of task-specific knowledge or strategies. In Experiment 1, practice effects were ostensibly attributable to participants adopting a qualitatively different strategy after their initial completion of the Pavlovian go/no-go task. Specifically, participants were able to exploit acquired knowledge of implicit dependencies between stimuli in the task to develop a process-of-elimination strategy that resulted in rapid learning and the attenuation of the desired choice biases. To address this issue, in Experiment 2 we redesigned the task to eliminate these dependencies and the formation of such a top-down strategy. This approach is consistent with previous research, whereby preventing participants from becoming aware of critical elements of a task design resulted in improved consistency and reliability of behavior, even with practice (McLean, Mattiske & Balzan, 2018). An important practical implication of these findings is that researchers seeking stable individual-difference measures should consider implementing a pre-baseline session protocol. Since parameters stabilize after the first session, conducting an initial familiarization session could effectively minimize initial learning biases when stable parameter estimates are needed.

It is important to note that although practice effects were reduced in our modified version of the Pavlovian go/no-go task, they were not eliminated altogether. Indeed, we observed smaller but still significant reductions in participants’ go and Pavlovian biases (with corresponding decreases in the approach bias model parameter) following the initial test session. For the purposes of individual-differences correlational research, these residual practice effects are tolerable because the reliabilities of the model parameters are still in an acceptable range. However, they may be worrisome for longitudinal studies where systematic changes in task performance are of interest (e.g., reduction in Pavlovian biases following psychotherapy (Geurts et al., 2022)). One possible solution might be increasing the length of the practice block, which was relatively brief in this study, and could be extended to help participants reach “steady state” performance prior to starting the actual task. Indeed, our results showed stability of performance on days 3 and 14, suggesting that task administrations after a longer practice may be usable for measuring changes in performance over the course of a mental health condition or treatment.

The current study has several notable limitations. We investigated the psychometric properties of two versions of the Pavlovian go/no-go task in a sample of online adult participants. The reliability of task measures, however, can vary as a function of the sample and the test setting. For example, previous research has shown that the reliability of a task completed by healthy adults can differ from that for adults with psychopathology (Cooper et al., 2017) or healthy children (Arnon, 2020). Importantly, our general sample of adult participants rated the modified Pavlovian go/no-go task as more mentally demanding than the original task (see Table S2). As such, our task may prove to be too challenging for other groups (e.g., children; patients) which may affect reliability. Future research is therefore necessary to validate the modified version of the task in other populations, or develop simplified variants of it.

A second limitation is that we only studied participants’ choice behavior. Previous studies have found that Pavlovian biases also manifest in response times (Algermissen et al., 2022; Millner et al., 2018), and these may be a meaningful index of individual differences (Betts et al., 2020; Millner et al., 2019; Scholz et al., 2020). Previous work also introduced a computational framework for jointly modeling participants’ choice and response time behavior on the task (Millner et al., 2019, 2018). This is notable because joint modeling of choice and response time had been found to improve the precision and reliability of parameter estimates from reinforcement learning models (Ballard & McClure, 2019; Shahar et al., 2019). As such, more research is warranted to investigate how the reliability of model-derived measures of behavior on the Pavlovian go/no-go task could be further improved by incorporating response times.

Limitations notwithstanding, our study demonstrates that it is possible to derive performance measures from the Pavlovian go/no-go task that are sufficiently reliable for use in individual-differences research. We encourage researchers to use and further adapt the modified version of the task presented here. In support of this goal, we have made all of our data and code publicly available (see Data and Code Availability statements).

## Data Accessibility Statement

The data that support the findings of this study are openly available on Github at https://github.com/nivlab/RobotFactory.

## Code Availability

All code for data cleaning and analysis associated with this study is available at https://github.com/nivlab/RobotFactory. The experiment code is available at the same link. The custom web-software for serving online experiments is available at https://github.com/nivlab/nivturk. A playable demo of the task is available at https://nivlab.github.io/jspsych-demos/tasks/pgng/experiment.html.

## Citation Diversity Statement

Recent work in several fields of science has identified a bias in citation practices such that papers from women and other minority scholars are under-cited relative to the number of such papers in the field (Bertolero et al., 2020; Dworkin et al., 2020). Here we sought to proactively consider choosing references that reflect the diversity of the field in thought, form of contribution, gender, race, ethnicity, and other factors. First, we obtained the predicted gender of the first and last author of each reference by using databases that store the probability of a first name being carried by a woman (Dworkin et al., 2020). By this measure (and excluding self-citations to the first and last authors of our current paper), our references contain 9.52% woman(first)/woman(last), 15.87% man/woman, 23.81% woman/man, and 50.79% man/man. This method is limited in that a) names, pronouns, and social media profiles used to construct the databases may not, in every case, be indicative of gender identity and b) it cannot account for intersex, non-binary, or transgender people. Second, we obtained predicted racial/ethnic category of the first and last author of each reference by databases that store the probability of a first and last name being carried by an author of color (Ambekar et al., 2009; Sood & Laohaprapanon, 2018). By this measure (and excluding self-citations), our references contain 3.82% author of color (first)/author of color(last), 12.98% white author/author of color, 16.75% author of color/white author, and 66.46% white author/white author. This method is limited in that a) names and Florida Voter Data to make the predictions may not be indicative of racial/ethnic identity, and b) it cannot account for Indigenous and mixed-race authors, or those who may face differential biases due to the ambiguous racialization or ethnicization of their names. We look forward to future work that could help us to better understand how to support equitable practices in science.

## Additional File

The additional file for this article can be found as follows:

10.5334/cpsy.127.s1 Supplementary materials. Figures S1 to S6 and Tables S1 to S7.
