# Supplementary material for: Improving the Reliability of the Pavlovian Go/No-Go Task for Computational Psychiatry Research
Source: Comput Psychiatr. 2025 Dec 18;9(1):231–52. doi: 10.5334/cpsy.127 (PMC12716264; doi:10.5334/cpsy.127)
Supplement: Supplementary materials. — Figures S1 to S6 and Tables S1 to S7. [file cpsy-9-1-127-s1.pdf]

Supplementary materials

Comparative stability of self-report measures

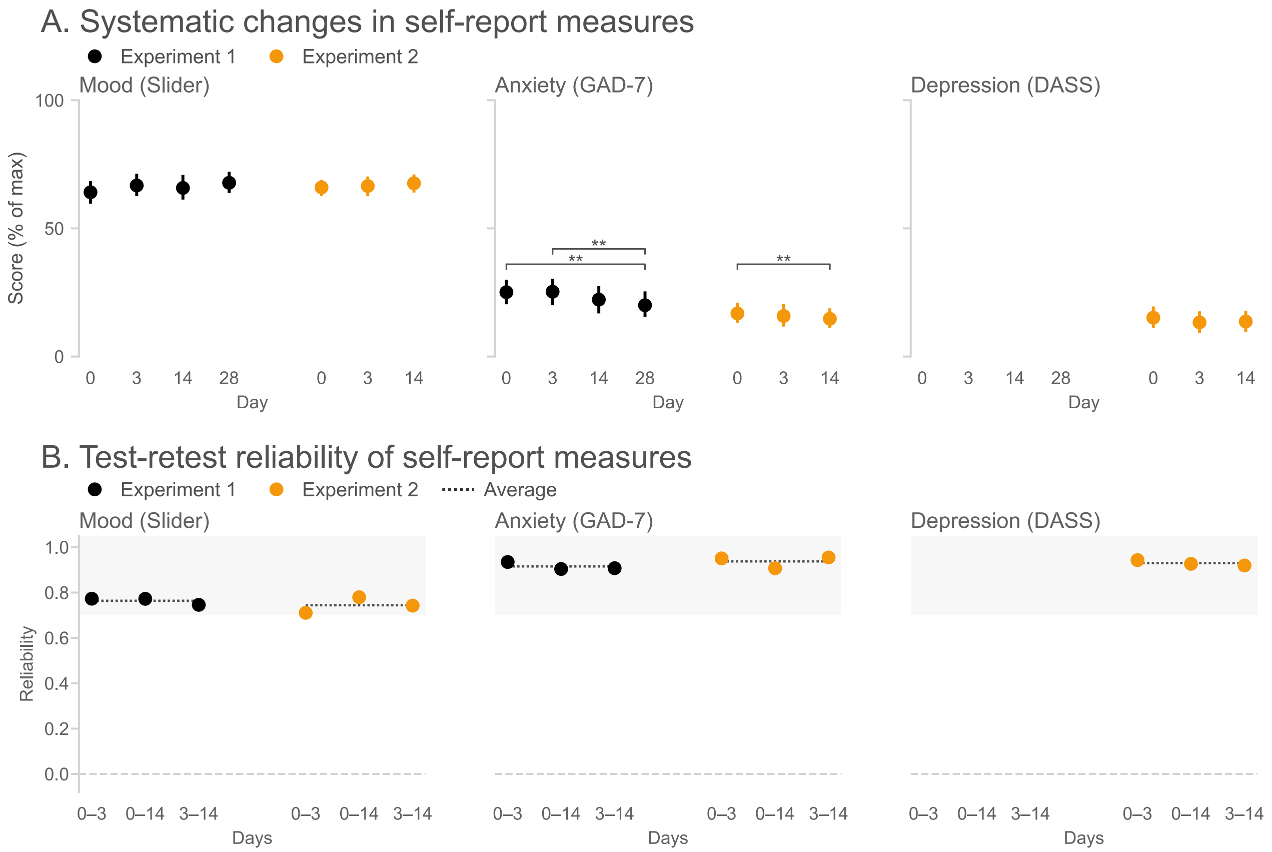

Figure S1: **Self-report measures suggest that mood and anxiety were relatively stable over the study period across participants.** (A) Group-level mood and symptom scores for each session. Error bars indicate 95% bootstrapped confidence intervals. ** Denotes significant pairwise difference ($p < 0.05$, corrected for multiple comparisons). (B) Test-retest reliability estimates for each self-report measure. Dotted lines indicate overall average. Shaded regions indicate conventional range of acceptable reliability ($\rho \geq 0.7$).

Example block of the modified Pavlovian go/no-go task

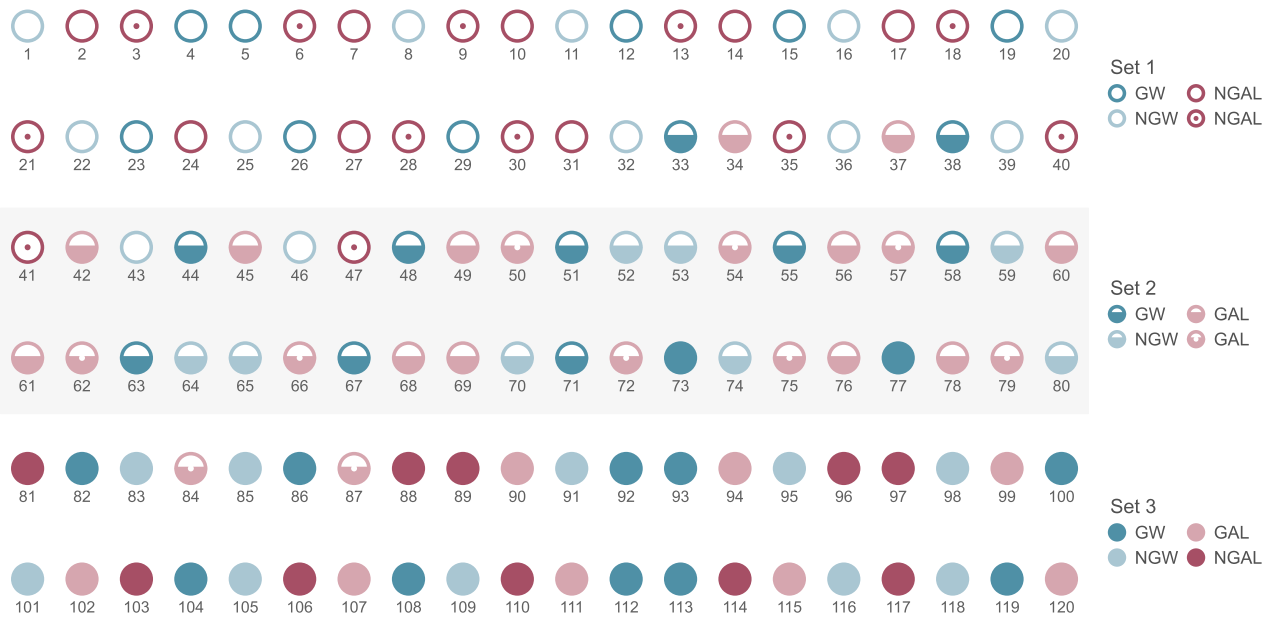

Figure S2: **Trial structure of an example block of the modified Pavlovian go/no-go task from Experiment 2**. In one task block, there were 12 unique stimuli (three of each trial type: go to win [GW; dark blue]; no-go to win [NGW; light blue]; go to avoid losing [GAL; light red]; no-go to avoid losing [NGAL; dark red]) divided into three sets (Set 1: empty circles; Set 2: half-filled circles; Set 3: filled circles). Each set was composed of approximately 40 trials (120 trials total). Each set, however, did not necessarily involve all four trial types. In this example block, Set 1 involved two NGAL stimuli and no GAL stimuli, and Set 2 involved two GAL stimuli and no NGAL stimuli.

Posterior predictive check for the best-fitting reinforcement learning model (Experiment 1)

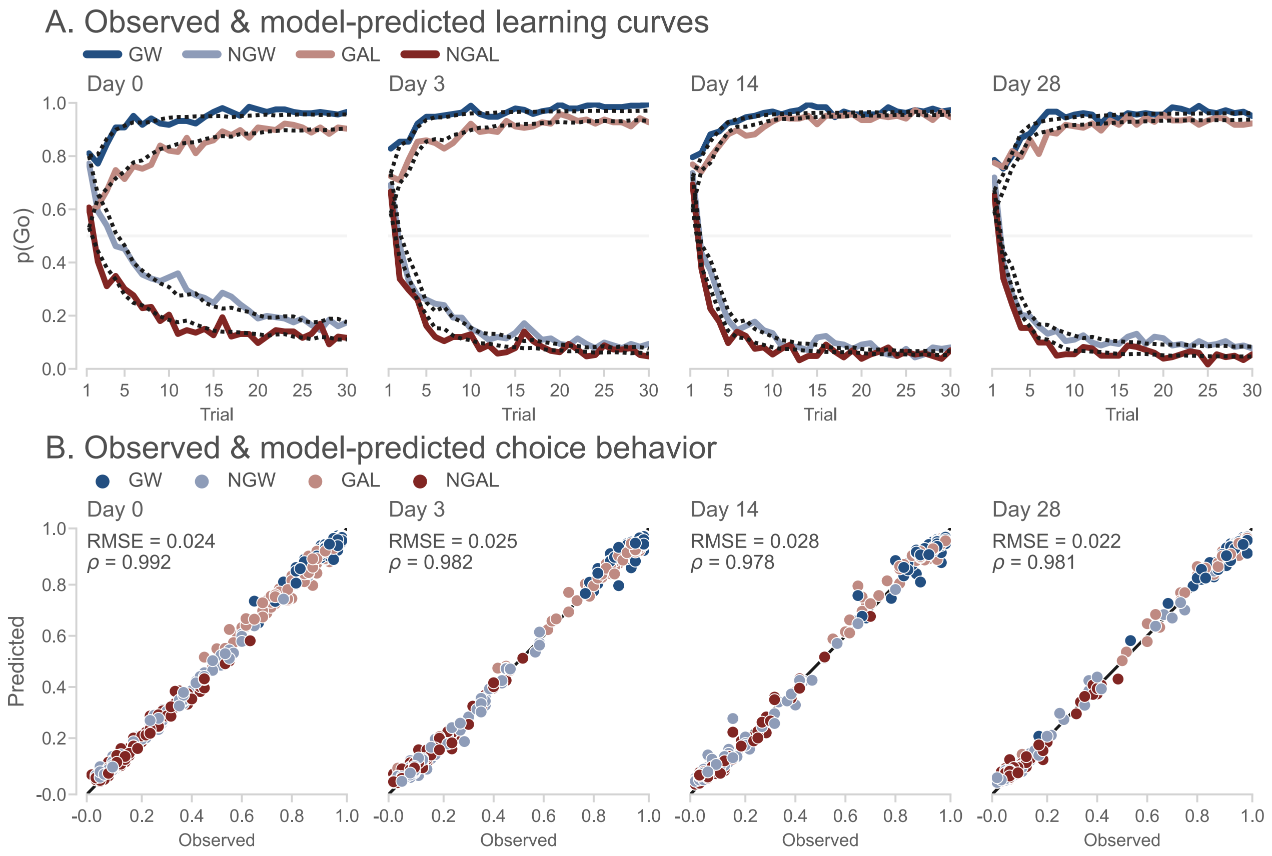

Figure S3: **Observed & model-predicted choice behavior for Experiment 1.** (A) Trial-by-trial choice behavior. Solid and dotted lines depict observed and model-predicted choice behavior, respectively. (B) Observed (x-axis) and model-predicted (y-axis) proportions of go responses for each trial type. Each point corresponds to one participant and condition. RMSE=root-mean-squared error between observed and model-predicted choice behavior. ρ: Spearman's correlation between observed and model-predicted choice behavior. All ρ's were exceptionally high at > 0.975.

Posterior predictive check for the best-fitting reinforcement learning model (Experiment 2)

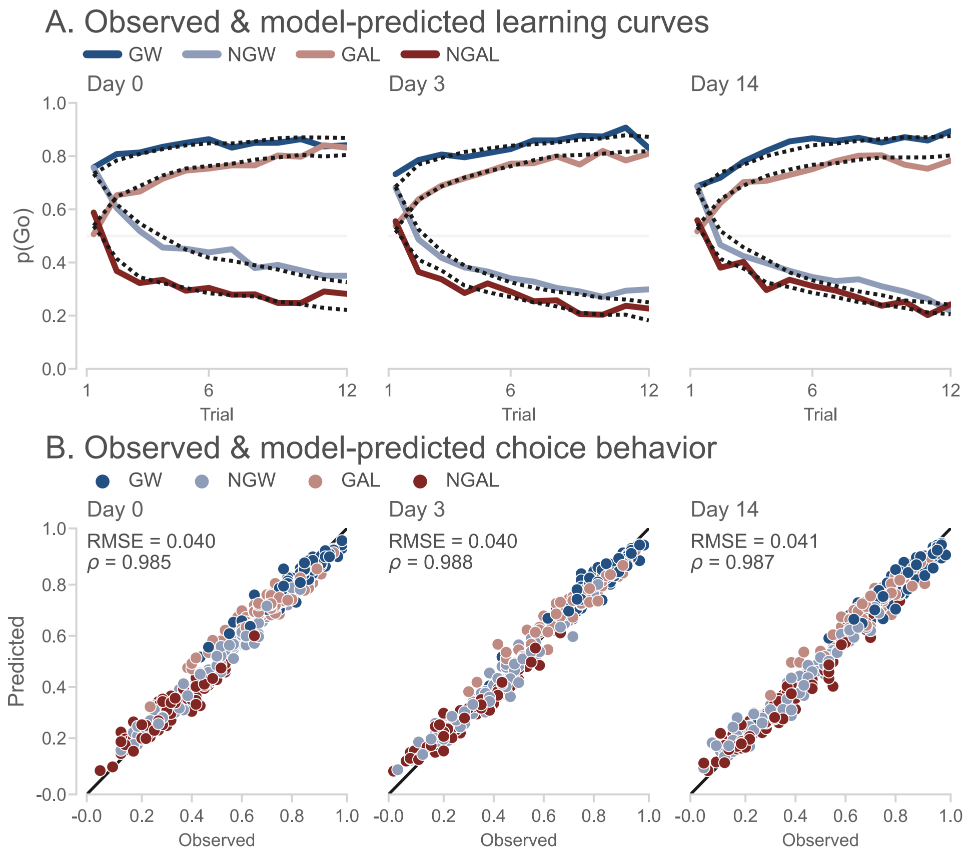

Figure S4: **Observed & model-predicted choice behavior for Experiment 2.** (A) Trial-by-trial choice behavior. Solid and dotted lines depict observed and model-predicted choice behavior, respectively. (B) Observed (x-axis) and model-predicted (y-axis) proportions of go responses for each trial type. Each point corresponds to one participant and condition. RMSE=root-mean-squared error between observed and model-predicted choice behavior. ρ: Spearman's rank correlation between observed and model-predicted choice behavior. Here, too, all ρ's were exceptionally high at > 0.985.

Split-half reliability of model parameters

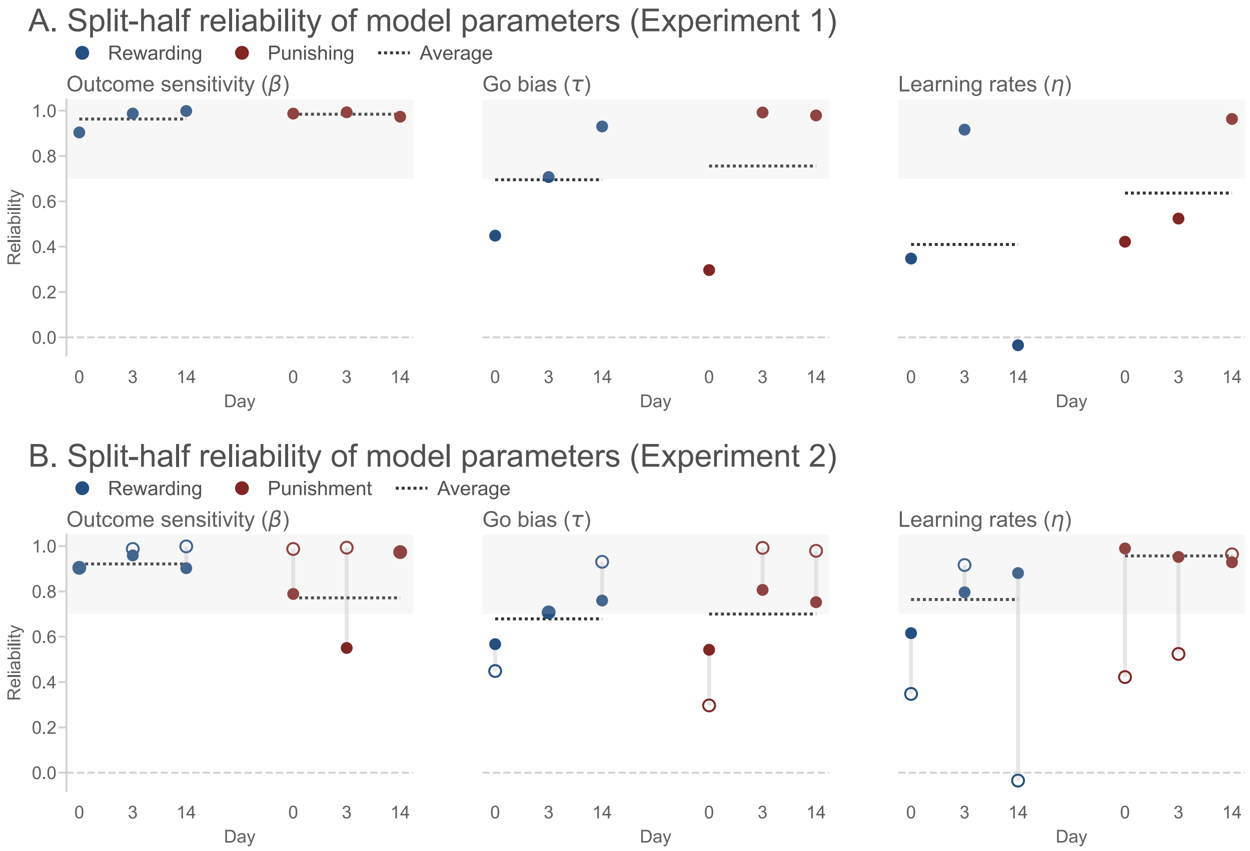

Figure S5: **Split-half reliability estimates for the best-fitting model parameters for (A) Experiment 1 and (B) Experiment 2.** Filled circles denote estimates for each experiment; open circles denote estimates from Experiment 1. Grey vertical lines show the change in reliability across experiments. Dotted lines indicate average reliability. Shaded regions indicate conventional range of acceptable reliability ($\rho \geq 0.7$).

Participant demographics

Variable	Experment 1 (N=103)	Experiment 2 (N=110)	p-value
**Gender**, N (%)			0.403
Men	55 (53.4%)	65 (59.1%)	
Women	47 (45.6%)	43 (39.1%)	
Transgender or nonbinary	1 (1.0%)	1 (0.9%)	
Rather not say	0 (0.0%)	1 (0.9%)	
**Age**, years			0.006
Mean (range)	35.5 (20–69)	39.6 (23–69)	
**Race & Ethnicity**, N (%)			0.264
White	80 (77.7%)	97 (83.6%)	
Black or African American	9 (8.7%)	10 (8.6%)	
Hispanic or Latino	9 (8.7%)	8 (7.3%)	
Asian	10 (9.7%)	3 (2.6%)	
American Indian/Alaska Native	0 (0.0%)	3 (2.6%)	
Rather not say	4 (3.9%)	3 (2.6%)	

Table S1: **Demographic characteristics of the participants in Experiments 1 and 2.** Participants could select more than one ethnic and racial identity. Therefore, participant counts and percentages in the corresponding section sum to more than 100%. The mean age of the two samples was compared via an independent samples t-test (df = 211, $\alpha = 0.05$, two-sided). The proportion of participants in each sample identifying as men or white was compared via the two sample proportions z-test (df = 211, $\alpha = 0.05$, two-sided). The two experiments differed significantly only in the age of participants, which was significantly older in Experiment 2.

Task appraisals

	Experiment	Day 0	Day 3	Day 14	Day 28
Difficulty	1	2.5 (1.1)	2.2 (0.9)	2.1 (1.0)	1.8 (0.8)
	2	3.3 (1.1)	3.0 (1.1)	3.1 (1.1)	-
Fun	1	3.9 (0.9)	4.0 (1.0)	3.9 (0.9)	4.1 (0.9)
	2	3.9 (0.9)	4.1 (0.8)	4.0 (0.8)	-
Clarity	1	4.8 (0.6)	4.9 (0.4)	5.0 (0.2)	4.9 (0.3)
	2	4.9 (0.5)	4.9 (0.2)	4.9 (0.2)	-

Table S2: **Mean (sd) of participants' ratings of task difficulty, fun, and clarity of the tasks.** All ratings were made on a 5-point Likert scale. Difficulty: "How difficult was the task?" (Very easy=1, Very hard=5); Fun: "How fun was the task?" (Very boring=1, Very fun=5); Clarity: "How clear were the instructions?" (Very confusing=1, Very Clear=5). Participants rated Experiment 2 as more difficult than Experiment 1.

Priors for Bayesian reinforcement-learning models

		Group-level	
Parameter	Participant-level	Mean	Standard deviation
Outcome sensitivity	$\beta_i \sim 10 \cdot \mathcal{N}(\mu_\beta, \sigma_\beta)$	$\mu_\beta \sim \mathcal{N}(0,1)$	$\sigma_\beta \sim$ Half-$t(3,0,1)$
Approach/avoidance bias	$\tau_i \sim 5 \cdot \mathcal{N}(\mu_\tau, \sigma_\tau)$	$\mu_\tau \sim \mathcal{N}(0,1)$	$\sigma_\tau \sim$ Half-$t(3,0,1)$
Learning rate	$\eta_i \sim \Phi\left(\mathcal{N}(\mu_\eta, \sigma_\eta)\right)$	$\mu_\eta \sim \mathcal{N}(0,1)$	$\sigma_\eta \sim$ Half-$t(3,0,1)$
Lapse rate	$\xi_i \sim \Phi\left(-2 + \mathcal{N}(\mu_\xi, \sigma_\xi)\right)$	$\mu_\xi \sim \mathcal{N}(0,1)$	$\sigma_\xi \sim$ Half-$t(3,0,1)$

Table S3: **Participant- and group-level priors specified for each parameter in the hierarchical Bayesian reinforcement-learning models.** Prior distributions incorporated task-specific assumptions: bimodal learning rate distributions to allow for participant heterogeneity and low lapse rates given quality-control exclusions. $\mathcal{N}$ denotes the Normal distribution, Φ denotes the cumulative density function for the standard normal distribution (used to constrain learning and lapse rates to be in the range $\in [0,1]$).

Complete descriptive statistics (Experiment 1)

	Within-session statistics					Between-session comparisons			
Variable	Day	Mdn	*d*	*p*	95% CI	Day 0	Day 3	Day 14	Day 28
Correct responses (%)	0	85.0	2.982	<0.001	[80.8, 87.9]	-			
	3	92.9	6.947	<0.001	[90.4, 94.6]	<0.001	-		
	14	94.6	10.310	<0.001	[93.1, 95.2]	<0.001	0.312	-	
	28	94.6	12.029	<0.001	[93.8, 95.8]	<0.001	0.659	0.986	-
Go bias (Δ%)	0	11.7	1.049	<0.001	[10.8, 13.3]	-			
	3	5.0	1.156	<0.001	[4.2, 5.8]	<0.001	-		
	14	4.2	1.124	<0.001	[2.9, 4.6]	<0.001	0.310	-	
	28	3.3	0.899	<0.001	[2.5, 4.2]	<0.001	<0.001	0.515	-
Valence bias (Δ%)	0	0.8	0.135	0.064	[-0.8, 2.5]	-			
	3	0.8	0.169	0.017	[0.0, 2.5]	0.992	-		
	14	0.0	0.000	0.264	[-0.8, 0.8]	1.000	0.807		
	28	0.0	0.000	0.257	[-0.8, 0.8]	0.851	0.851	1.000	-
Pavlovian bias (Δ%)	0	9.2	1.237	<0.001	[6.7, 11.7]	-			
	3	1.7	0.450	<0.001	[0.8, 2.5]	<0.001	-		
	14	1.7	0.450	<0.001	[0.8, 2.5]	<0.001	0.808	-	
	28	0.8	0.225	0.001	[0.0, 1.7]	<0.001	0.270	0.998	-
Feedback sensitivity (Δ%)	0	9.4	1.250	<0.001	[7.2, 11.5]	-			
	3	4.5	0.770	<0.001	[3.4, 6.2]	<0.001	-		
	14	3.0	0.581	<0.001	[1.8, 4.7]	<0.001	0.841	-	
	28	2.8	0.704	<0.001	[2.2, 4.3]	<0.001	0.180	0.630	-

Table S4: **Within- and between-session descriptive statistics for Experiment 1.** Between-session values are p-values for the pairwise comparisons. Mdn=median.

Model comparison by session (Experiment 1)

Session	Model	Accuracy (%)	PSIS-LOO	ΔPSIS-LOO
Day 0	1	82.2	-37241.1	-2050.3 (42.9)
	2	84.1	-38327.4	-964.0 (30.7)
	3	85.2	-38992.9	-298.5 (17.7)
	4	85.2	-39145.6	-145.8 (11.1)
	5	85.3	-39129.9	-161.5 (8.9)
	6	85.4	-39190.3	-101.1 (6.7)
	7	85.6	-39291.4	-
Day 3	1	88.7	-38446.8	-1468.0 (34.0)
	2	90.0	-38979.3	-935.5 (27.5)
	3	90.8	-39492.8	-422.0 (17.7)
	4	90.8	-39644.9	-269.9 (12.9)
	5	90.8	-39629.8	-285.0 (12.3)
	6	90.9	-39666.1	-248.7 (11.6)
	7	91.0	-39914.8	-
Day 14	1	90.1	-38350.9	-928.6 (26.5)
	2	91.5	-38777.0	-502.5 (19.1)
	3	91.5	-38971.4	-308.1 (14.7)
	4	91.7	-39066.7	-212.8 (11.6)
	5	91.7	-39072.2	-207.3 (11.3)
	6	91.8	-39093.8	-185.7 (10.8)
	7	91.9	-39279.5	-
Day 28	1	89.4	-37419.1	-1155.6 (30.7)
	2	91.0	-37928.1	-646.6 (23.5)
	3	92.1	-38360.7	-214.1 (11.8)
	4	92.1	-38404.5	-170.3 (9.3)
	5	92.0	-38434.0	-140.7 (8.5)
	6	92.1	-38451.6	-123.1 (7.6)
	7	92.3	-38574.7	-

Table S5: **Model comparison broken down by session for Experiment 1.** Accuracy: trial-level choice prediction accuracy between observed and model-predicted Go responses. PSIS-LOO: approximate leave-one-out cross-validation presented in deviance scale (smaller numbers indicate better fit). ΔPSIS-LOO: difference in LOO values between each model and the best fitting model (M7).

Complete descriptive statistics (Experiment 2)

	Within-session statistics					Between-session comparisons		
Variable	Day	Median	d	p	95% CI	Day 0	Day 3	Day 14
Correct responses (%)	0	67.5	1.828	<0.001	[65.8, 69.0]	-		
	3	71.7	2.063	<0.001	[69.6, 74.6]	0.003	-	
	14	69.6	1.441	<0.001	[66.5, 73.8]	0.146	0.335	-
Go bias (Δ%)	0	19.2	1.108	<0.001	[15.0, 22.5]	-		
	3	13.3	0.899	<0.001	[10.0, 15.0]	0.007	-	
	14	14.2	0.882	<0.001	[7.5, 17.5]	0.003	0.382	-
Valence bias (Δ%)	0	-2.5	0.289	0.002	[-4.2, 0.8]	-		
	3	0.8	0.096	0.245	[-0.8, 3.3]	0.096	-	
	14	2.5	0.337	0.004	[0.0, 4.2]	0.002	0.851	
Pavlovian bias (Δ%)	0	12.5	1.065	<0.001	[10.0, 15.0]	-		
	3	8.3	0.843	<0.001	[6.7, 10.0]	0.027	-	
	14	7.5	0.867	<0.001	[5.0, 9.2]	0.027	0.997	-
Feedback sensitivity (Δ%)	0	28.2	2.254	<0.001	[25.7, 30.9]	-		
	3	29.4	2.142	<0.001	[24.7, 32.0]	0.632	-	
	14	26.8	2.267	<0.001	[24.1, 29.4]	0.942	0.461	-

Table S6: **Within- and between-session descriptive statistics for Experiment 2.** Between-session values are p-values for the pairwise comparisons.

Model comparison by session (Experiment 2)

Session	Model	Accuracy (%)	PSIS-LOO	ΔPSIS-LOO
Day 0	1	71.5	-33517.7	-2746.3 (48.6)
	2	75.4	-35145.3	-1118.8 (33.1)
	3	77.3	-36002.4	-261.6 (16.8)
	4	77.2	-36074.8	-189.2 (12.0)
	5	77.4	-36117.1	-147.0 (10.7)
	6	77.4	-36124.5	-139.6 (9.1)
	7	77.6	-36264.1	-
Day 3	1	74.0	-31023.5	-1656.0 (38.0)
	2	77.4	-31996.2	-683.3 (26.0)
	3	78.1	-32447.1	-232.5 (15.7)
	4	78.2	-32513.7	-165.9 (11.3)
	5	78.1	-32508.9	-170.7 (10.6)
	6	78.3	-32543.9	-135.7 (9.1)
	7	78.7	-32679.6	-
Day 14	1	73.3	-31265.0	-1802.8 (39.2)
	2	76.8	-32474.5	-593.4 (24.8)
	3	77.4	-32833.5	-234.3 (16.4)
	4	77.3	-32833.9	-233.9 (13.1)
	5	77.6	-32893.1	-174.7 (11.9)
	6	77.6	-32880.4	-187.5 (11.3)
	7	78.1	-33067.8	-

Table S7: **Model comparison broken down by session for Experiment 2.** Accuracy: trial-level choice prediction accuracy between observed and model-predicted Go responses. PSIS-LOO: approximate leave-one-out cross-validation presented in deviance scale (smaller numbers indicate better fit). ΔPSIS-LOO: difference in PSIS-LOO values between each model and the best fitting model (M7).

Reaction Time Analysis

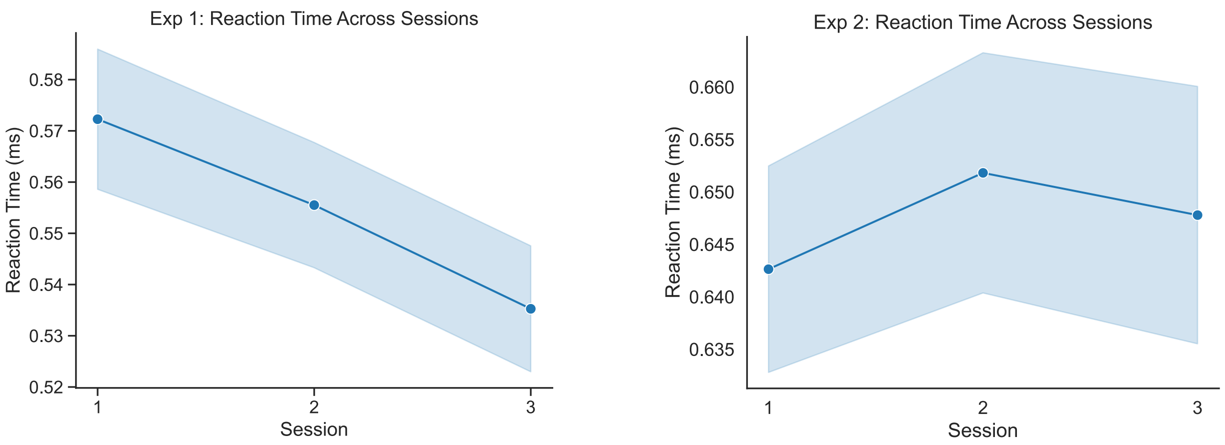

Figure S6: **Reaction time patterns differ between experiments, reflecting distinct practice effects.** Reaction times (RT) for Go trials across sessions. Left: Experiment 1 demonstrates a significant linear decrease across sessions (Session 1: $M = 0.572s$, Session 2: $M = 0.556s$, Session 3: $M = 0.535s$; linear trend: $t(88) = -3.652$, $p < 0.001$), with significant pairwise decreases from Session 1 to Session 3 ($t(88) = 3.652$, $p = 0.001$, $d = 0.347$) and Session 2 to Session 3 ($t(88) = 2.667$, $p = 0.014$, $d = 0.181$). Right: Experiment 2 shows stable RTs across sessions (Session 1: $M = 0.643s$, Session 2: $M = 0.652s$, Session 3: $M = 0.648s$) with no significant linear trend ($t(90) = -0.422$, $p = 0.674$) or pairwise differences (all $p > 0.05$ after FDR correction). These findings indicate that improved performance in Experiment 1 was accompanied by faster, more automatic responses rather than strategic slowing, while stable RTs in Experiment 2 align with reduced practice effects.
